# Metabolomic and Metataxonomic Fingerprinting of Human Milk Suggests Compositional Stability over a Natural Term of Breastfeeding to 24 Months

**DOI:** 10.3390/nu12113450

**Published:** 2020-11-11

**Authors:** Natalie S. Shenker, Alvaro Perdones-Montero, Adam Burke, Sarah Stickland, Julie A.K. McDonald, Kate Alexander-Hardiman, James Flanagan, Zoltan Takats, Simon J.S. Cameron

**Affiliations:** 1 Department of Surgery and Cancer, Imperial College London, London W12 0HS, UK; natalie.shenker09@imperial.ac.uk (N.S.S.); j.flanagan@imperial.ac.uk (J.F.); 2 Department of Metabolism, Digestion and Reproduction, Imperial College London, London SW7 2AZ, UK; a.perdones-montero@imperial.ac.uk (A.P.-M.); a.burke@imperial.ac.uk (A.B.); sarahstickland9@gmail.com (S.S.); julie.mcdonald@imperial.ac.uk (J.A.K.M.); k.hardiman@imperial.ac.uk (K.A.-H.); z.takats@imperial.ac.uk (Z.T.); 3 MRC Centre for Molecular Bacteriology and Infection, Imperial College London, London SW7 2AZ, UK; 4 Institute for Global Food Security, School of Biological Sciences, Queen’s University Belfast, Belfast BT9 5DL, UK

**Keywords:** human milk, metabolomic fingerprinting, metataxonomics

## Abstract

Sparse data exist regarding the normal range of composition of maternal milk beyond the first postnatal weeks. This single timepoint, observational study in collaboration with the ‘Parenting Science Gang’ citizen science group evaluated the metabolite and bacterial composition of human milk from 62 participants (infants aged 3–48 months), nearly 3 years longer than previous studies. We utilised rapid evaporative ionisation mass spectrometry (REIMS) for metabolic fingerprinting and 16S rRNA gene metataxonomics for microbiome composition analysis. Milk expression volumes were significantly lower beyond 24 months of lactation, but there were no corresponding changes in bacterial load, composition, or whole-scale metabolomic fingerprint. Some individual metabolite features (~14%) showed altered abundances in nursling age groups above 24 months. Neither milk expression method nor nursling sex affected metabolite and metataxonomic fingerprints. Self-reported lifestyle factors, including diet and physical traits, had minimal impact on metabolite and metataxonomic fingerprints. Our findings suggest remarkable consistency in human milk composition over natural-term lactation. The results add to previous studies suggesting that milk donation can continue up to 24 months postnatally. Future longitudinal studies will confirm the inter-individual and temporal nature of compositional variations and the use of donor milk as a personalised therapeutic.

## 1. Introduction

Human infants, as altricial mammals, are born with relatively immature central nervous, immune and intestinal systems, requiring high care input [1]. Lactation is a core evolved adaptation unique as a reproductive strategy to all mammals [2]. Human milk is a complex biofluid containing thousands of bioactive molecules acting individually and in synchrony to drive and programme the normal development of each organ system [3,4,5,6]. Recent discoveries in the field of the microbiome, gut–brain axis development and essentiality of human milk provision for normal development have highlighted the critical nature of human milk consumption over at least the first year of life [7,8,9,10,11,12]. Given that the evolutionary pressures that drove species-specific milk composition resulted from enhanced immunity and antimicrobial function, with nutrition a relatively recent secondary function, it is unsurprising that the normal duration of breastfeeding by mothers living in cultures untouched by the Western lifestyle is at least 2 years, and in societies unaffected by Westernisation continues until children reach immunological maturity [13,14].

Remarkably, scant literature exists on human milk composition beyond the early postnatal weeks, with only two recent studies examining milk composition over 1 year [15,16]. However, the complexity of milk is vast, as highlighted by recent proteome and lipidome profiling studies [17,18], and is likely to be greater as regards the functionality of molecules operating individually and in combination with others. The deficit in understanding of whether and how human milk changes over a normal course of lactation prevents parents from making informed decisions regarding infant feeding, contributes to public and medical misapprehensions of the role of human milk in development, and has led to non-evidence-based age cut-offs, usually of 6 months, for the recruitment of new milk donors to human milk banks.

As a highly complex biofluid, human milk requires a wide repertoire of analytical tools to ensure experimental coverage. As a result of resource and sample limitations, studies into human milk are typically limited to a portion of the available analytes. Frequent analytes for coverage fall within the general areas of genomics [19] and transcriptomics [20], requiring sequencing technologies, and proteomics [18], metabolomics [21], and lipidomics [17], which are heavily dependent on mass spectrometry. In this study, we provide assessments of the metabolic fingerprint of human milk using high-throughput rapid evaporative ionisation mass spectrometry (REIMS) and of the metataxonomic composition using 16S rRNA gene amplicon sequencing from mothers breastfeeding nurslings between 3 months and 4 years. REIMS is a particularly promising technique for the study of human milk as it does not require sample preparation or extraction. Thus, the analysis of samples can take place with reduced bias resulting from factors including storage, extraction, and chromatography [22], and give a metabolic ‘snapshot’ that may be more reflective of the natural state of the sample.

To the best of our knowledge, this is the first study to look to examine human milk over this age range using this combination of analytical techniques, allowing the semi-quantitative analysis of fatty acids, complex lipids, and small molecular weight metabolites, and the microbial profiling of human milk. Uniquely, it is also co-designed with members of the citizen-science group ‘The Parenting Science Gang’, who were involved in the study design and execution. Using this unique approach, we show that the macro-level metabolic and microbial composition of human milk is maintained between 3 and 24 months of nursling age, with variations in small numbers of metabolite features observed as the nursling age increases beyond 24 months.

## 2. Materials and Methods

This study was co-created with members of the Parenting Science Gang, who assisted with the preparation of experimental design, recruitment of participants, preparation of study documents including the participant lifestyle questionnaire and informed consent forms, and the logistics of sample collection. Detailed experimental methodology is provided in the accompanying Supporting Information document.

### 2.1. Participant Recruitment

Members of the Parenting Science Gang were recruited as participants in this study through online social media groups. Participants were self-selecting based on the reported length of breastfeeding, with a minimum nursling age of 3 months. All participants provided written informed consent prior to sample donation and all information was link anonymised before analysis. No limitations were placed on participants with regards to lifestyle choices prior to study participation. Sample collection for this study was carried out as a sub-project (approval number R18006) of the Breastmilk Epigenetic Cohort Study under the Imperial College Healthcare Tissue Bank (HTA licence 12275), which received ethical approval from the Wales Regional Ethics Committee (reference 17/WA/0161). All procedures were carried out in accordance with the ethical standards of the Helsinki Declaration of the World Medical Association. Link anonymised lifestyle questionnaires were completed prior to sample donation. Sections requesting lifestyle information were based on self-reported declarations of intake and not on a validated food frequency questionnaire.

### 2.2. Sample Collection and Handling

All sample collection was completed on the same day at Charing Cross Hospital, Imperial College London, London, UK. Participants were allocated to hourly donation groups between 09:00 and 16:00 h. During the donation session, participants were asked to express the entire contents of either or both breasts using either hand expression or a manual (various supplied by participants) or electric pump (Central Medical Supplies, Leek, UK). Prior to use, manual and electric pumps were heat-sterilised using at-home, consumer-available microwave sterilisation bags. No restrictions were placed on the participants during the donation period, including the feeding of children, and no sterilisation of the breast was completed. After expression, samples were transferred to sterile containers and transported to the analysis laboratory in an adjacent building. Pre-made, bovine-derived, commercially available formula milk for ages up to 6 months, 6 to 12 months, and 12 to 24 months were purchased from local supermarkets and processed in the same way as human milk samples for subsequent analysis. Unprocessed human milk samples were treated as Hazard Group 2 material and handled within class two microbiological safety cabinets using standard laboratory personal protective equipment. For laser assisted-REIMS (LA-REIMS) analysis, a Class IVb CO_2_ laser was used for sample heating within a modified class two microbiological safety cabinet which had been adapted with suitable containment precautions and matched to the wavelength of the laser. All chemicals and solvents were handled in accordance with the material data safety sheet provided by their manufacturer.

### 2.3. Sample Processing and Total Volume and Fat Determination

Within 30 min of donation, samples were vortex mixed and split into three aliquots: 2 mL for metabolic fingerprinting, 2 mL for DNA extraction, and the remaining used for total volume and fat determination in the required number of 50 mL centrifuge tubes for total volume. Metabolic fingerprinting samples were analysed within 2 h of donation, as detailed below. Sample volumes for DNA extraction were stored at −80 °C until processed, as detailed below. Remaining volumes were stored at +4 °C until the day after collection for total volume and fat determination, after which they were stored at −80 °C. Total sample volume was determined through volumetric calculation using graduated 50 mL centrifuge tubes and the total of aliquot milk for metabolic fingerprinting and DNA extraction. Total fat determination was completed through centrifugation of milk samples in 15 mL centrifuge tubes at 3000× *g* for 5 min at +4 °C. The volume of the total fat layer was visually determined and a percentage against total volume was calculated. Where a participant had donated samples from both breasts, the sample from the breast with the largest total volume was used in comparison of total volume and fat levels, for REIMS metabolic fingerprinting, and for metataxonomic analysis. Nursling age groups were determined as 3 to 6 months (12 participants), 6 to 12 months (12 participants), 12 to 24 months (16 participants), 24 to 36 months (14 participants), and 36 to 48 months (8 participants).

### 2.4. Total DNA Extraction from Human Milk Samples and Metataxonomic Analysis

Each 2 mL aliquot of human milk underwent centrifugation for 10 min at 14,000× *g*, after which the fat layer was removed through use of a sterile cotton swab. The supernatant was then removed through aspiration and the pellet carried forward for DNA extraction using a FastDNA™ SPIN kit for soil (MP Biomedical, Irvine, CA, USA) following the manufacturer’s recommended protocol except that bead beating was carried in three cycles at speed setting 6.0 with cooling on ice for 2 min between cycles. Extracted DNA was eluted into 50 µL of DNase-free water. The concentration of extracted dsDNA was determined using the Qubit dsDNA broad range assay kit (ThermoFisher Scientific, Loughborough, UK) and a Qubit Fluorimeter 4.0 (ThermoFisher Scientific) with 1 µL of eluted DNA. DNA extraction batches consisted of 23 samples and one extraction negative control using 2 mL of PCR grade Water (Roche Diagnostics, Mannheim, Germany) in place of milk volume. Copy number for the 16S rRNA gene in each sample was determined using qPCR against gene copy number standards created through amplification of the full 16S rRNA gene of five randomly selected samples, as previously reported [23]. For metataxonomic analysis, DNA extracts were normalised to 5 ng/µL and sample libraries were created through amplification of the V1 to V2 region of the 16S rRNA gene as previously described [24]. Detailed information on bioinformatic analysis of sequences, and statistical analysis is provided in the Supplementary Information.

### 2.5. Metabolic Fingerprinting Using Laser Assisted Rapid Evaporative Ionisation Mass Spectrometry (LA-REIMS)

Within 1 h of sample donation, 2 mL of milk samples were placed into a 24 well tissue culture plate (Greiner Bio-One, Frickenhausen, Germany) and underwent centrifugation at 3000× *g* for ten min in a +4 °C chamber to produce a layer suitable for LA-REIMS analyses. Samples were analysed in donation batches so not all plate wells were filled in all runs. Each plate was placed on a raised platform within a Freedom Evo 75 (TECAN, Mannedorf, Switzerland) robot liquid handler adapted for high-throughput analysis, as previously reported [25,26]. Sample heating and evaporation utilised a helium-cooled 10.6 µM wavelength CO_2_ laser with fibre optic beam guide (FELS-25A, OmniGuide, Lexington, KY, USA) which was focused to a 500 µM spot size using a lens focusing system (Aesculight, Bothell, WA, USA). Detailed information on REIMS analysis, instrument operating parameters, spectral processing, and statistical analysis is provided in the Supplementary Information.

### 2.6. Correlation Analysis of Multi-Omic Datasets

Data matrices for REIMS metabolite fingerprinting (total ion count normalised), metataxonomics (removal of samples with less than 1000 reads and normalised for percentage abundance at operational taxonomic unit (OTU) level), and lifestyle variables were imported into the R environment (version 3.5.3) using R Studio (version 1.2.1335). Pearson correlations were calculated for all variable combinations with a significance cut-off of *p* value less than 0.05 after Bonferroni correction and correlation strength cut-off of less than −0.5 or more than 0.5. Subsequent plots were constructed using the ggplot2, magrittr, and ggpubr packages. To remove potential false positive correlations for metataxonomic and metabolomic fingerprinting comparisons, significant correlations between features and OTUs were removed from further analysis if the OTU was not present in more than 10% of samples.

### 2.7. Statistical Analysis

Detailed information on statistical analysis for metataxonomic and metabolic fingerprinting is provided in the supporting information and is specific to the online analysis pipeline used. Statistical parameters used in correlation analysis of multi-omic datasets is given in Section 2.6. For analysis of participant phenotypic and demographic data in Table 1, data were imported into Prism 8 software (Version 8.1.2, GraphPad Software, San Diego, CA, USA). An Anderson–Darling normality test with a *p* value threshold of less than 0.05 was used to determine the appropriate univariate statistical test for quantitative variables and either a one-way ANOVA or Kruskal–Wallis with *p* value threshold of less than 0.05. Chi Square tests were used for categorical variables if they met the assumptions of the test—namely that all expected values are greater than 1 and at least 20% of the expected values are greater than 5. For expressed human milk volume, fat percentage, and estimation of bacterial load data, a non-normal distribution was observed for datasets using an Anderson–Darling normality test with the *p* value threshold of less than 0.05. As such, a Kruskal–Wallis test was used with a *p* value threshold of less than 0.05 with class differences identified using a post hoc Dunn’s multiple comparisons test with an adjusted *p* value threshold of less than 0.05.

### 2.8. Availability of Raw Data

Raw 16S rRNA amplicon gene sequencing data are available through the European Nucleotide Archive under accession number PRJEB35510. Raw mass spectrometry data files are available through the MetaboLights repository under accession number MTBLS1370. Individual link anonymised participant information is available as Supplementary Data Matrix 1.

## 3. Results and Discussion

Expressed human milk samples were collected during an 8-h period from 62 breastfeeding mothers with nurslings between 3 months and 4 years of age; each sample was processed within 1 h of expression. Samples were expressed using either a single electric pump (Ameda Elite; Central Medical Supplies, UK) or hand-expressed with the support of a lactation consultant. Table 1 shows generalised demographic and lifestyle information for mothers based on age of nursling and milk expression method with Supplementary Data Matrix 1 showing anonymised individual characteristics. No significant group differences were observed based on demographic, lifestyle differences, or physical attributes (one-way ANOVA *p* value above 0.05 for quantitative characteristics with normal distribution, Kruskal–Wallis *p* value above 0.05 for quantitative characteristics with non-normal distributions, and Chi Square *p* value > 0.05 for categorical characteristics which met the assumptions of the test) for nursling age groupings. Data for ethnicity and diet did not meet the assumptions of the test and would not, therefore, provide robust results. Therefore, it cannot be excluded that these differences may have impact on the observed results.

### 3.1. Nursling Age Correlates Only with Expression Volume

**Figure 1:**
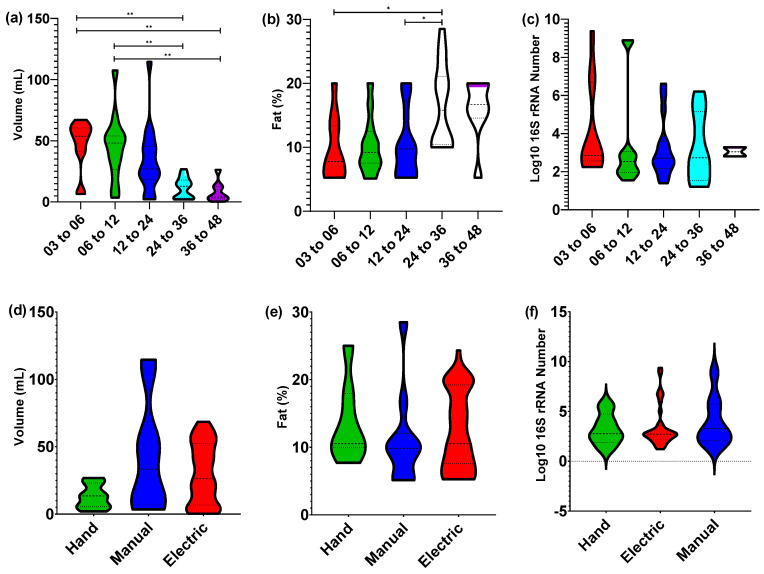
Nursling age and expression method effects volume but not fat or bacterial load of milk. Total expressed volume of human milk for (**a**) age groups and (**b**) milk expression method; total fat percentage—0% to 100%—of human milk for (**c**) age groups and (**d**) milk expression method; and Log_10_ of 16S rRNA gene copy number as a measure of total bacterial count for (**e**) age groups and (**f**) milk expression method. All datasets show a non-normal distribution based on an Anderson–Darling normality test with *p* value less than 0.05. Only significantly different groups (*p* value less than 0.05), as determined by a Kruskal–Wallis test, were used with a *p* value threshold of less than 0.05 with class differences identified using a post hoc Dunn’s multiple comparisons test with an adjusted *p* value threshold of less than 0.05. *p* value significance thresholds identified as * <0.05, ** <0.01. A total of 62 human milk replicates were used for expressed volume and fat percentage comparisons, and 49 for Log_10_ of 16S rRNA number comparisons.

Investigating the total volume, percentage of fat, and microbial load of human milk samples showed that significant differences were only present for expression volume after the nursling age exceeded 24 months (Kruskal–Wallis with post hoc Dunn’s multiple comparisons test with *p* value < 0.01; Figure 1). Although breast pump expression of human milk is not a direct measure of breastfeeding volume [27], it is likely that the reduction in expression volume beyond 24 months of nursling age is a result of a reduction in breastfeeding frequency. Investigation of the macronutrient components of human milk have previously identified as significant, but low-level correlations, between lactation beyond 12 months and total fat, protein, and carbohydrate levels when analysing single timepoint samples [16]. However, when multiple longitudinal samples were taken from a cohort of women breastfeeding from 11 to 17 months postpartum, no significant alterations in carbohydrates or fat were observed [15]. Within this single = timepoint collection cohort, we observed a single significant difference between the fat percentage between 3 to 6 months and 24 to 36 months (Kruskal–Wallis with post hoc Dunn’s multiple comparisons test with *p* value < 0.05). This was not observed between the 3 to 6 months and the 36 to 48 months age groups. Further, there was no significant difference observed between the detected 16S rRNA gene copy number, which is a proxy for bacterial load. This finding is particularly important with regards to the microbial load within human milk, as an increase may present an immunological burden on the infant and pose issues with regards to suitability for human milk banking.

### 3.2. Core bacterial Microbiome Exists across Lactation Period

**Figure 2:**
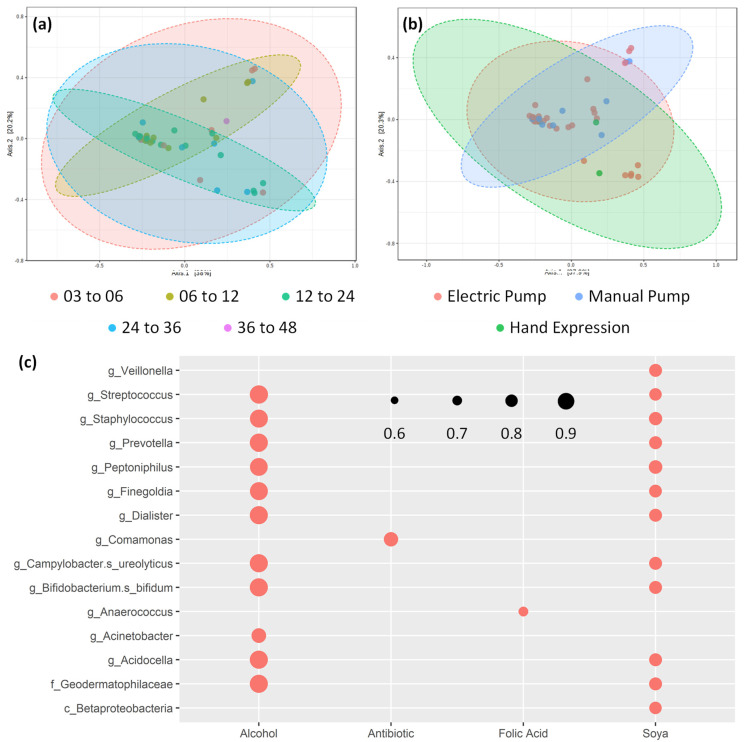
Microbiome fingerprinting shows influence of lifestyle factors but not nursling age. Metataxonomics using amplicon sequencing of the 16S rRNA gene shows no significant separation (PERMANOVA *p* value above 0.05) in beta diversity for (**a**) age of nursling nor (**b**) expression method using principal coordinate analysis. Shading shows 95% confidence intervals of groupings. Additionally, (**c**) lifestyle factors were correlated with microbiome features at the finest taxonomic resolution achieved. Size of dot indicates size of correlation and only positive correlations were significant, with *p* value below 0.05. A total of 46 human milk replicates were used in this analysis.

A total of 46 participants’ samples were suitable for 16S rRNA gene amplicon sequencing, Supplementary Table S1. The reduction from the total participant number was a result of sample volume, with a minimum of 5 mL being needed in each sample to conduct REIMS metabolic fingerprinting and DNA extraction. After quality filtering, samples contained an average of 7180 read counts (range from 1042 to 31,673) from a total of 217 OTUs. Negative extraction and sequencing controls were compared to all samples (Supplementary Figure S1) and formed a significantly distinct cluster outside of the confidence intervals of the experimental samples. The issue of contaminated DNA extraction kits is now well known within the field [28]. However, it appears to be an issue primarily in samples with low microbial biomass, which was not applicable to these human milk samples. Recent work specifically applied to extraction of human milk samples has shown that differences in DNA quantity and metataxonomic profiles can occur based on chosen methodology. Our study used a bead beating DNA extraction kit, which was shown to give the highest quality of extracts based on 260/280 ratios [29]. In line with previous studies, we identified a core genus-level microbiome within human milk samples, which was dominated by *Streptococcus* and *Rothia* [19,30,31], with other genera in lower level abundance including *Actinomyces*, *Acinetobacter*, *Veillonella*, *Granulicatella*, *Burkholderia*, *Sediminibacterium*, and *Corynebacterium*. A large portion of reads remained as taxonomically unassigned, which suggests that a high degree of currently undefined species may be present within the human milk microbiome. Multivariate principal coordinate analysis of sample groupings based on nursling age shows no separation at OTU level abundance (Figure 2a) based on overlap of shaded 95% confidence intervals. Univariate analysis for individual OTUs showed no significant differences driven by nursling age after correction for multiple hypothesis testing. We completed further correlation analysis using Pearson’s correlation coefficient to identify co-occurring clusters of microbiome features (Supplementary Figure S2). Limited by the observational nature of this study, it is not possible to determine whether there is a biological reason for these co-occurring microbiome features, but a potential avenue of further study is offered, to explore whether these correlations continue into the gut microbiome or are linked to features of human milk composition.

Human milk had been long considered sterile but is now known to contain a complex of microorganisms that act as a foundation on which the infant gut microbiome develops. The origins of the human milk microbiome are still not fully understood, but are likely to be a combination of translocation from the mother’s gut microbiome and transference from the infant’s oral microbiome [30]. The development of DNA sequencing technologies has allowed the study of microorganisms beyond those that are culturable under traditional laboratory conditions [32]. Substantial temporal variation has been reported in the human milk microbiome between colostrum and mature milk (after 1 month) [19]. As a result of the large compositional variation in human milk during transition from colostrum to a mature supply, we did not include samples from mothers with nurslings below 3 months in age. As a ‘snapshot’ study, we do not observe any significant change in the human milk microbiome between 3 months and up to 48 months of nursling age. However, a trend in human milk microbiome studies appears to be the high degree of individual differences [19,30,31] which may mask longitudinal changes. Therefore, a longitudinal cohort study that samples human milk from birth through lactation beyond 6 to 12 months would be required to definitively confirm that the microbiome is indeed stable beyond 3 months of nursling age.

### 3.3. Detection of Metabolite Features in Human Milk Using LA-REIMS

**Figure 3:**
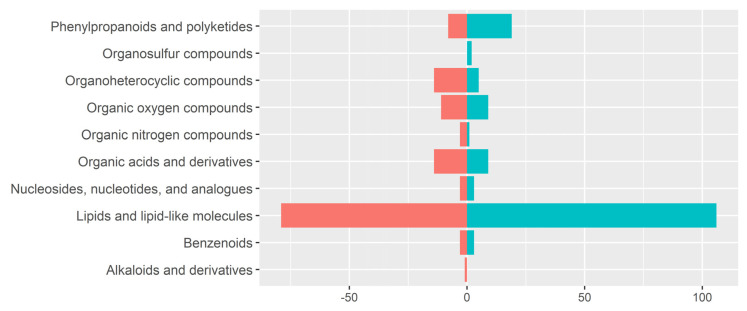
Super-class classification of chemical taxonomy of detectable metabolite features using REIMS. A total of 386 metabolites features were detected (210 in negative ion detection mode and 176 in positive ion detection mode) across all human milk samples. Using database searches and an accuracy threshold of <10 ppm, 293 features across both modalities were identified, Supplementary Figure S4a. Super-class level of chemical taxonomy using the Human Metabolome Database system is shown, with features detected negative ion detection mode shown in pink (**left**) and positive ion detection mode in blue (**right**). Class level identifications are shown in Supplementary Figure S4b.

Research exploring the composition of human milk has typically focussed on the macro-level components such as total carbohydrate, protein, and fat. Although these features are important in evaluating human milk as a total energy source, many of the biochemical and immunological impacts that human milk may have on the nursling are likely to be driven by the micro-level components, including metabolites, specific fatty acids, and complex lipids. Here, we utilised the high-throughput, sample-preparation-free laser-assisted rapid evaporative ionisation mass spectrometry (LA-REIMS) to obtain a metabolomic fingerprint of human milk samples within 1 h of expression from 62 participants. As this study presents the first use of REIMS for the analysis of human milk, we also analysed commercially available, pre-made bovine-derived formula. Multivariate analysis of the metabolomic fingerprint of formula alongside human milk shows that clear and significant separation is evident between the two (Supplementary Figure S3). From this, REIMS can be shown to be both a useful tool for the metabolomic fingerprinting of a sample, as differences between human and bovine-derived formula are observed, and also as a robust method, as very tight clusters of formula milk samples, which are replicates of a pre-formulation, are shown. To determine the range of metabolite features detectable in human milk using LA-REIMS, we completed database searches for tentative identifications within a mass accuracy threshold of 10 ppm. A total of 386 metabolites features were detected (210 in negative ion detection mode and 176 in positive ion detection mode) across all human milk samples, and 293 features were identified (Supplementary Figure S4a). Chemical taxonomy classifications to the super-class level are given in Figure 3 and show that most identified features are lipids, and lipid-like molecules in both modalities. Classifications at the sub-class and direct-parent level show that triacylglycerides are dominant in positive ion detection mode and a combination of glycerophospholipids and amino acids and peptides in negative ion detection mode. Of note is the tentative detection of human milk oligosaccharides and bile acids, which are important factors in the development of the infant gut microbiome.

### 3.4. Metabolomic Fingerprinting Indicates Subtle Shifts beyond 2 Years

**Figure 4:**
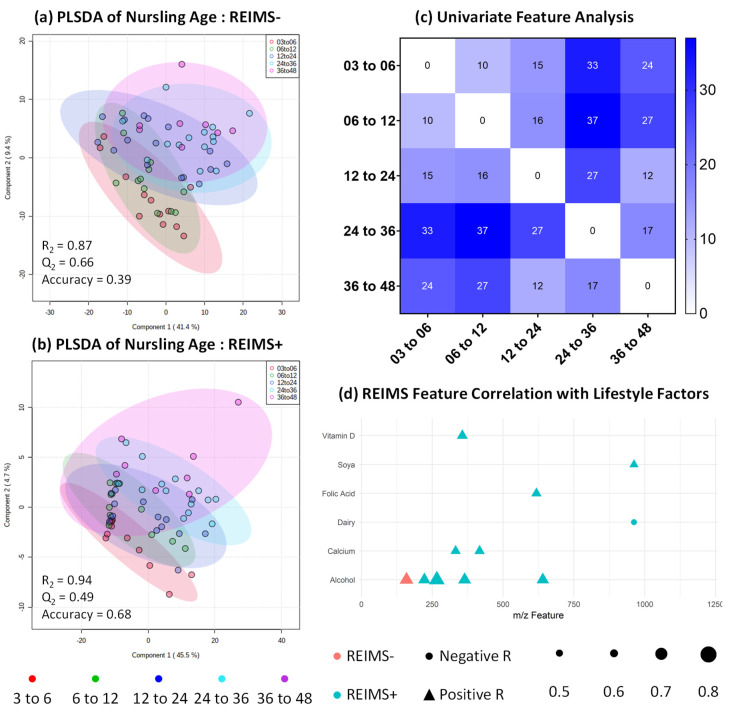
Significantly different univariate components identified in negative ion detection mode. Partial least squares-discriminant analysis (PLS-DA) modelling of nursling age for both (**a**) negative ion detection and (**b**) positive ion detection modes show no significant separation, but (**c**) univariate ANOVA shows significant (False discover rate (FDR) corrected *p* value below 0.05) differences for nursling age between defined age groups, across both rapid evaporative ionisation mass spectrometry (REIMS) ion detection modes, with the greatest identifiable differences between age groups either side of 24 months. Further analysis of (**d**) Pearson’s correlation coefficient analysis against reported lifestyle factors shows several significant correlating features with a *p* value threshold of below 0.05 and correlation coefficient cut-off of above 0.5 for positive correlations or below −0.5 for negative correlations. Colour coding indicates REIMS ion detection modality. Data point shape indicates whether correlation coefficient is positive or negative and size indicates strength of correlation coefficient. A total of 62 human milk replicates were used.

Negative and positive ion detection modes were used in REIMS metabolomic fingerprinting analysis and handled as separate datasets. For both polarities, multivariate modelling using partial least square discriminant analysis (PLSDA) showed no significant separation between nursling age groups (Figure 4a,b). However, univariate analysis using one-way analysis of variance with correction for multiple hypothesis testing identified significant differences in both modalities (Figure 4c), with eight features in negative and 36 features in positive ion detection modes significantly different between at least two nursling age groups. This is equal to 3.8% of the total detected features in negative ion detection mode and 20.4% in positive ion detection mode. Tentative identifications of the significantly different features are given in  Supplementary Figure S5, the majority of which appear to be very long chain fatty acids or complex lipids. Dipeptides and metabolites associated with dietary intake, such as flavonoids, are also tentatively identified. We completed literature searches for all tentatively identified significantly different metabolites and did not find any which were associated with potential toxicity or harm. The majority (82%) of inter-group significant differences were between nursling age groups above and below 24 months, with only 14% occurring between groups above and below 12 months. Univariate results suggest a trend where significant differences result from higher abundance in older nursling age groups (Supplementary Figure S5). This may be because of a concentration of human milk content due to the reduced expression volumes for nursling age groups above 24 months (Figure 1a). As this is the first report of the metabolomic fingerprints of human milk beyond 12 months, there are minimal comparisons that can be made with previously reported results.

The metabolomic exploration of human milk is a developing area. Work to date has focussed on the metabolomic profiling of human milk and differences between milk produced by mothers of pre-term and to-term nurslings, typically using nuclear magnetic resonance spectroscopy [21,33,34,35]. Here, we present the metabolomic fingerprinting of the widest nursling age range in the current literature. Current breastfeeding guidelines recommend exclusive breastfeeding to 6 months, continued alongside the introduction of solids for 2 years [36]. This is supported by our findings in that there is no significant change over the 4-year age range that we studied, but there are significant changes in some fingerprint features beyond 2 years of nursling age. These primarily include changes in complex lipids, which are the main detectable components of human milk using the LA-REIMS method. Previous work has suggested that the energy and fat levels of human milk are significantly increased beyond one year of nursling age [37], although we did not observe significant differences in total fat percentage in our cohort. In recent years, some evidence has emerged on similar patterns with regards to protein concentration of human milk beyond the first year of breastfeeding. A hypothesised role for tight junction regulation has been proposed [38], which can be affected by various factors including weaning. There is limited evidence for this with regards to the lipid composition of human milk but is an area that could elucidate a mechanism for observed changes over a natural term of breastfeeding. It should, however, be noted that the mass spectrometry method that we employed, in line with all other analytical approaches [22], is only able to analyse a distinct range of analytes within human milk. REIMS can detect metabolites, fatty acids, and complex lipids but not some major components of human milk, such as complex or intact proteins. These are an important with regards to development of the infant gut immune system, and it may be that their contribution to human milk composition varies over the studied nursling age reported here, but, due to analytical limitations, was not measurable.

### 3.5. Potential Confounding Variables on Human Milk Composition

Three different expression methods were used by participants: hand expression, manual expression pump, and electric expression pump. Participants were free to choose whichever method they were most comfortable using. Total expressed volume of human milk was significantly higher when an expression pump was used (*p* < 0.001 for manual pump and <0.05 for electric pump; Figure 1), but this did not correspond with a change in either total fat content or 16S rRNA gene copy number. Metataxonomic composition analysis showed no significant differences between expression method using modelling of beta-diversity (Figure 2b). Furthermore, no significant differences were observed with regards to multivariate PLSDA modelling of REIMS metabolic fingerprinting in either negative or positive ion detection modes (Supplementary Figure S6a,b). Nursling gender was also analysed using PLSDA modelling (Supplementary Figure S6c,d), with no significant differences detected. Univariate statistical analysis was also conducted for both expression method and nursling gender (data not shown), with no statistically significant result observed at FDR corrected *p* value threshold below 0.05. Although scant evidence is available, similarity between metabolic fingerprints of human milk from different expression methods is not unexpected. Here, we used the ambient ionisation technique REIMS to acquire a metabolic fingerprint of expressed samples within 1 h. The lack of sample storage and preparation associated with the method is likely to minimise any sample degradation because of microbial activity. Further, participants in this study all fed nurslings at the breast and expression via hand or pump was not the typical method of lactation. Although it may be possible for long-term expression method to impact the metabolome of human milk, within this participant cohort it is not considered a factor that could bias interpretation of changes associated with nursling age. Differences in microbial content of human milk expressed via different pumping methods has been previously shown; primarily with regards to cultured pathogenic bacteria [39,40,41], where insufficient contamination of pumps and tubing has taken place. Within this study, we used a recommended at-home microwave sterilisation method which has been shown to reduce microbial load of expressed milk to that of hand-expressed milk [42].

### 3.6. Human Milk Shows Compositional Stability against Self-reported Lifestyle and Dietary Factors

To assess the extent to which individual differences may affect human milk composition, we performed a correlation analysis between self-reported lifestyle factors and metabolomic fingerprint features detected with REIMS. Using a significance threshold of below 0.05 after Bonferroni correction and a correlation coefficient cut-off of below −0.5 or above 0.5, we identified one feature in negative ion and 11 in positive ion mode (Figure 4d). Across both modalities, the largest contributing lifestyle factors were reported intake of units of alcohol, albeit only for five features. The remaining correlating features were associated with either dietary intake of soya or dairy, or intake of supplements including Vitamin D, folic acid, and calcium. It should be noted that one participant self-reported a weekly intake of 40 units of alcohol (Supplementary Data Matrix 1), which is considerably higher than the rest of the cohort. This high single value may be responsible, to some extent, for the identification of false positive correlations and, therefore, the link between alcohol and human milk composition should be explored further in a larger cohort that can provide sufficient statistical power for higher alcohol intake groups. Considering the range of detectable features using REIMS and the number of lifestyle factors explored, the lack of significantly correlating fingerprint features suggests that human milk shows compositional stability against lifestyle and dietary factors. Analysis for metataxonomic correlations shows a similar relationship between intake of alcohol and soya and microbiota features, with 11 and 12 correlating features, respectively. The apparent stability of human milk nutritional composition in relation to lifestyle factors is in line with previously reported work, although maternal dietary intake, particularly in terms of lipid source, appears to have a major impact on the availability of fatty acids in human milk [43,44]. Within this experimental group, we also explored frequency of soya, vegetable, fruit, meat, fish, eggs, and dairy intake but found no significantly correlated features. It should be noted that this analysis was based on self-reported intake and was not collected using a validated dietary questionnaire.

### 3.7. REIMS Shows Potential Co-Occurrence of Microbiota and Metabolite Features

**Figure 5:**
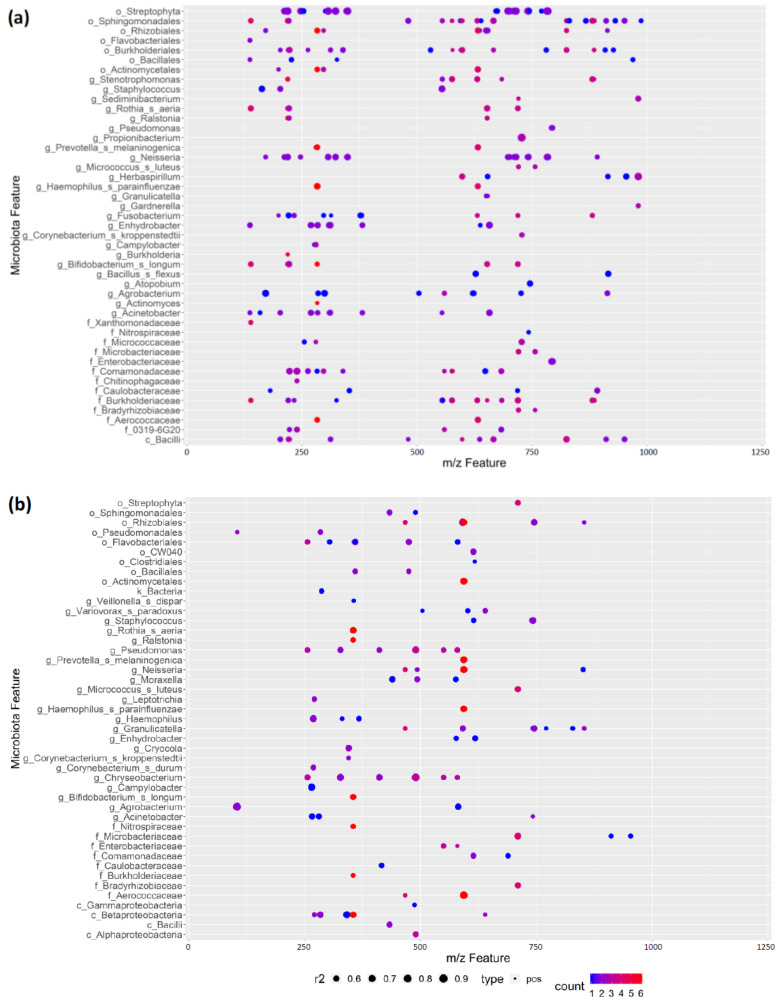
Significant positive correlations between rapid evaporative ionisation mass spectrometry (REIMS) and microbiome features. Pearson’s correlation coefficient was used to identify significant correlations (*p* value below 0.05) between microbiome and metabolomic features using (**a**) negative ion detection mode and (**b**) positive ion detection mode REIMS data. Microbiome features are shown to finest taxonomic resolution achieved on y-axis and REIMS features as a mass-to-charge ratio on x-axis. Size of data point indicates strength of correlation feature and colour indicates the total number of significant correlations for that REIMS feature against microbiome features. Only positive correlations were identified in this analysis. A total of 46 human milk replicates are represented in this figure.

Long considered a sterile biofluid, the microbial composition of human milk is a fundamental building block of the developing nursling microbiome [45,46]. Additionally, human milk contains prebiotic compounds, such as human milk oligosaccharides, which are important in establishing the infant microbiome [47]. We wanted to further explore our dataset and determine whether there are co-occurrences of microbiota and metabolite features within our human milk samples, which may indicate evolutionary pressures. To minimise false positive correlations because of low abundance features, we used a cut-off criterion of the OTU being present in at least 10% of samples. Figure 5 shows the presence of a high number of positively correlating REIMS features with OTUs derived from 16S rRNA gene sequencing. For both negative and positive ion detection modes, features across the acquired 50 to 1200 *m/z* mass range were strongly correlated. Of note is the detection of correlations between the tentatively identified neutral human milk oligosaccharide lacto-N-tetraose and the *Neisseria* genus, of which many non-pathogenic species are found within the human gut microbiome. This may suggest that this oligosaccharide acts as an energy source to help the establishment of commensal *Neisseria* species. However, there is only evidence of the role of human milk oligosaccharides in disrupting mucosal adhesion in the literature [48], and therefore, their function in promoting non-pathogen *Neisseria* species colonisation will require further research. Although correlations in an observational study such as this limit the scope of the conclusions that can be drawn, this finding suggests REIMS has the analytical sensitivity and resolution to be used as a rapid profiling tool to further explore this area in future longitudinal studies. Of particular interest is that many of the identified metabolites are not human milk oligosaccharides, Supplementary Data Matrix 2, which raises the potential that there is a wider role for human milk components, such as complex lipids, in the establishment of the newborn microbiome and potential inhibition of pathogen adhesion to the mucosal surface.

### 3.8. Implications for Human Milk Banking

Globally, human milk bank numbers are increasing, with over 700 now operating in more than 60 countries [49]. The majority of human milk banks target donor recruitment to mothers with infants aged less than 6 months, on the understanding that their milk will be more closely matched to the needs to premature infants. However, this current work and that of other groups [15] suggest that there is no change and a possible increase in overall fat content, the source of approximately 50% of calories in human milk [50]. In addition, with increasing age of infant, no increase in bacterial load was detected, which is an important aspect for donor milk safety and the likelihood that donated milk will pass pre-pasteurisation microbiological tests [51]. Further work will be required to understand the relationship between dietary intake and milk composition for milk donors. These current findings lend support to mothers donating milk up to 2 years postnatally, but further longitudinal studies will be required to address interindividual changes over a full term of lactation. Additionally, future studies will be required to investigate whether donor milk from mothers with older nurslings may be more appropriate for very low birth weight infants who often require nutritional supplementation.

## 4. Limitations

This study did not include analysis of milk from mothers with babies aged under 3 months, nor did it include mothers with pre-term babies. We further did not include infant characteristics such as term of birth, birth weight, or postnatal growth trajectory in our analysis. Analysis of these dyads and infant characteristics will be important for future profiling studies. Furthermore, this was a single-point-in-time analysis, which may obscure inter- and intra-individual variability in human milk composition. The sample group in this study was self-selecting from members of the Parenting Science Gang, and as data on education level or household income were not collected, it is not possible to determine how representative the sample group is of the wider population. However, the results form the basis for a future longitudinal recruitment of mothers, which will be able to not only answer questions around composition over a natural term of lactation, but also inform future milk banking guidelines for donor recruitment. As well as the highly abundant bioactive lipid metabolites, human milk also contains over 200 oligosaccharides, which together are critical for the establishment of innate and adaptive immunity [6,52,53,54], neurodevelopment [55,56], and the development of a normal diverse neonatal gut microbiome, rich in *Lactobacillus* and *Bifidobacteria* species. Future studies will aim to broaden the profiling of samples to include more oligosaccharides and proteome profiling, alongside other functional molecular groups.

## 5. Conclusions

Public health messaging has largely focussed on promoting and protecting exclusive breastfeeding for up to 6 months [57], while the WHO’s recommendations to breastfeed for 2 years and beyond have been marginalised [36]. Mainstream attitudes and perceptions in the general public and healthcare professionals are that human milk loses nutritional quality after 6 months, and that the avoidance of formula is only essential in resource-poor environments without assured access to clean water. As recent studies have shown though, an aberrant gut microbiome as a result of non-exclusive human milk infant feeding acts as a missing link between genetic risk and environmental triggers, implying that infant feeding across human societies has broader ramifications for public health than previously understood [7,10,58,59]. The current study suggests stability in human milk composition in terms of both metabolomic and microbiome composition for up to 24 months. Further studies to understand human milk composition and function should be conducted. Parents need this information to help them make decisions, milk banks need to be able to understand the variability of donated milk for the nutrition of premature and sick babies, and public policy bodies need information about milk composition to drive societal support mechanisms that enable mothers to meet their breastfeeding goals.

## Figures and Tables

**Table 1:** Participant demographic information.

	Age Group						Expression Method			
	03 to 06	06 to 12	12 to 24	24 to 36	36 to 48	*p* Value	H	M	E	*p* Value
**Total Participants**	12	12	16	14	8	N/A	7	10	45	N/A
**Age (Years)**	33.75 (3.41)	34.75 (2.80)	32.81 (4.90)	35.29 (5.55)	37.63 (5.76)	0.24 ^‡^	35.86 (4.53)	33.60 (5.68)	34.56 (4.56)	0.77 ^‡^
**Ethnicity**										
Caribbean	0	0	0	1	1	N/A *	0	1	1	N/A *
Chinese	0	0	1	0	1	N/A *	0	0	2	N/A *
Other	1	0	0	0	0	N/A *	0	1	0	N/A *
White	11	12	12	12	5	N/A *	6	8	38	N/A *
White Irish	0	0	1	0	0	N/A *	0	0	1	N/A *
White Mixed	0	0	1	1	1	N/A *	1	0	2	N/A *
White Other	0	0	1	0	0	N/A *	0	0	1	N/A *
**Body Mass Index**										
Pre-Pregnancy	24.57 (4.99)	27.21 (4.83)	23.95 (4.22)	26.14 (4.30)	24.21 (4.24)	0.32 ^†^	25.96 (4.86)	26.09 (4.33)	24.92 (4.61)	0.70 ^†^
Post-Pregnancy	25.79 (4.60)	27.82 (4.82)	25.01 (3.92)	26.98 (5.42)	26.27 (5.13	0.59 ^†^	26.34 (5.68)	26.66 (3.87)	26.23 (4.81)	0.97 ^†^
**Previous Pregnancies**	1.58 (0.79)	1.50 (0.52)	1.38 (0.62)	1.57 (0.93)	1.50 (0.93)	0.95 ^‡^	1.57 (0.98)	1.50 (0.53)	1.49 (0.73)	0.95 ^‡^
**Sex of Nursling**										
Female	6	8	8	5	3	0.57 *	2	6	22	0.44 *
Male	6	4	8	9	5		5	4	23	
**Diet**										
Meat Eater	11	10	16	9	6	N/A *	6	8	38	N/A *
Pescetarian	1	0	0	2	1		0	1	3	
Vegetarian	0	2	0	3	1		1	1	4	

Summary of participants present in each of five age groups and three expression method groups. Values are given either as counts or mean of group with corresponding standard deviation in brackets. For group count values, *p* value is given as output of Chi-Square test (marked with *) where the data format meets the assumptions of the test. Where this is not the case, the *p* value is given as N/A and no conclusions can be drawn on significant differences between groups. For numerical values, class normality was tested using the Anderson–Darling normality test with *p* value threshold below 0.05. For normally distributed data, the *p* value is given as the outcome of one-way ANOVA (marked with ^†^). For non-normally distributed data, the *p* value is given as the outcome of Kruskal–Wallis (marked with ^‡^).

## Supplementary Materials

The following are available online at https://www.mdpi.com/2072-6643/12/11/3450/s1, Figure S1: Negative extraction controls show no biologically meaningful contamination, Figure S2: Correlation analysis of microbiota features shows co-occurring clusters, Figure S3: REIMS is capable of clear differentiation between human and bovine-derived formula, Figure S4: Chemical taxonomy classification of identified REIMS metabolite features, Figure S5: Univariate statistics suggest significant differences may result from concentration, Figure S6: PLSDA modelling of expression method and nursling gender as potential confounders, Supplementary Data Matrix 1: Individual link anonymised participant information, and Supplementary Data Matrix 2: Tentative identification of LA-REIMS detected metabolite features.
