# Supplementary material for: Metabolomic and Metataxonomic Fingerprinting of Human Milk Suggests Compositional Stability over a Natural Term of Breastfeeding to 24 Months
Source: Nutrients. 2020 Nov 11;12(11):3450. doi: 10.3390/nu12113450 (PMC7697254; doi:10.3390/nu12113450)
Supplement: Supplementary file 1 [file nutrients-12-03450-s001.zip › psg_nursling_age_nutrients_revision_esi_v1.docx]

**Metabolomic and Metataxonomic Fingerprinting of Human Milk Suggests Compositional Stability Over a Natural Term of Breastfeeding to 24 Months**

Natalie S Shenker^1^, Alvaro Perdones-Montero^2^, Adam Burke^2^, Sarah Stickland^2^, Julie AK McDonald^2,3^, Kate Alexander-Hardiman^2^, James Flanagan^1^, Zoltan Takats^2,4^, Simon JS Cameron^4*^.

1 Department of Surgery and Cancer, Imperial College London, UK.

2 Department of Metabolism, Digestion and Reproduction, Imperial College London, UK.

3 MRC Centre for Molecular Bacteriology and Infection, Imperial College London, London, UK

4 Institute for Global Food Security, School of Biological Sciences, Queen’s University Belfast, UK.

* Corresponding Author: Simon JS Cameron, s.cameron@qub.ac.uk

**Contents**

**Methods** Supplementary methods to support manuscript Experimental Section

**Figure S1** Comparison of negative DNA extraction controls to experimental samples

**Figure S2** Correlation analysis of microbiota features shows co-occurring clusters

**Figure S3** REIMS is capable of clear differentiation between human and bovine-derived formula

**Figure S4** Chemical taxonomy classification of identified REIMS metabolite features

**Figure S5** Univariate statistics suggest significant differences may result from concentration

**Figure S6** PLSDA modelling of expression method and nursling gender as potential confounders

**References** Supporting references

**Supplementary Methods**

**Estimation of Bacterial Load using 16S rRNA Quantitative Polymerase Chain Reaction**

PCR for each selected sample was completed in 30 µL reaction volumes consisting of 15 µL of 2 x BioMix (BioLine, London, UK), 1.2 μL each of 27f (5′-AGA GTT TGA TCC TGG CTC AG-3′) and 1492r (5′- GGT TAC CTT GTT ACG ACT T-3′) primers to give a final concentration of 500 nM, and 1.8 µL of neat DNA extract with the remaining volume made up to with 10.8 µL of PCR grade water (Roche Diagnostics, Mannheim, Germany). PCR cycling conditions consisted of an activation step at 95°C for 5 minutes followed by 35 cycles of denaturation at 94°C for 30 seconds, annealing at 55°C for 30 seconds and elongation at 72°C for 1 minute with a final elongation step at 72°C for 10 minutes. Amplified products were verified on a 1.0% agarose gel run in 0.5% Tris-borate-EDTA (TBE) buffer and purified using the AMPure XP system (Beckman Coulter, Brea, USA). Pooled samples were quantified using the Qubit dsDNA broad range assay kit as described above and the total number of 16S rRNA gene copies determined. Serial dilutions of 10^10^, 10^9^, 10^8^, 10^7^, 10^6^, 10^5^, 10^4^, 10^3^, 10^2^, 10^1^, 10^0^ and 10^-1^, were made using PCR grade water for subsequent qPCR reactions. Quantitative PCR was carried out in 20 µL reaction volumes containing 10 µL SYBR green mix (Applied Biosystems Life Technologies, Carlsbad, USA), 2 µL of extracted DNA, 1 µL each of EUBF1 (5′-GTG STG CAY GGY TGT CGT CA-3′) and EUBR1 (5′-ACG TCR TCC MCA CCT TCC TC-3′) primers to give a final concentration of 400 nM, and 6 µL of PCR grade water (Roche). The qPCR run used a StepOne Plus (Applied Biosystems Life Technologies) instrument and consisted of an activation step at 95°C for 10 minutes and then 40 cycles of denaturation at 95°C for 15 seconds and annealing at 60°C for 1 minute. At the end of each cycle the fluorescence of each well was recorded. A melt curve was completed after the amplification cycle to confirm uniform amplicon formation and ran in a gradient from 95°C to 60°C in 0.3°C increments, with each held for 15 seconds. Negative controls were run in each plate where neat DNA was replaced with PCR grade water. Standards were run in duplicate for each plate and each sample was run in duplicate over separate instrument runs. Estimates of 16S rRNA gene copy number in each sample was calculated against calibration curves in the StepOne Plus Software (Version 2.3, Applied Biosystems Life Technologies) against the calibration standard curve for each plate. Means of duplicate were calculated for each sample and data presentation and statistical analysis using one-way ANOVA with *post-hoc* Tukey’s test, corrected for multiple comparisons, was completed in Prism 8 software (Version 8.1.2, GraphPad Software, San Diego, USA).

**Metataxonomic Profiling using 16S rRNA Amplicon Gene Sequencing of V1 to V2 Region**

A SequalPrep Normalization Plate Kit (Life Technologies) was used for clean-up and normalisation of the index PCR. The NEBNext Library Quant Kit for Illumina (New England Biolabs, Ipswich, USA) was used for sample library quantification. Sequencing employed the MiSeq platform (Illumina Inc, San Diego, USA) using the MiSeq Reagent Kit (version 3.0, Illumina) and paired-end 300 bp chemistry. All negative controls for DNA extraction batches were included in amplicon sequencing library preparation. The resulting sequences were analysed using the QIIME 2 pipeline[1]. Demultiplexed paired-end sequences were merged using the DADA2[2] function with the forward and reverse sequences truncated to 250 bp based on quality scores determined using FastQC[3]. Sequences underwent denoising, filtering, trimming, and chimera removal using DADA2. The taxonomic assignment of the consensus operational taxonomic unit (OTUs) sequences was determined through classification against the Greengenes database (version 13.8)[4] using the open-reference vsearch at 97% sequence identify function. MicrobiomeAnalyst[5] was used for statistical analysis employing a low count minimum filter of 2 and prevalence in more than 10% of samples, and a low variance filter of 10% of features to remove based on inter-quantile range. Negative extraction control samples and those samples with less than 1000 read total were removed prior to rarefaction normalisation to the sample with the lowest number of reads. Relative log expression was used for data transformation. Thresholds for core microbiome taxa were set at prevalence in at least 10% of samples and with a relative abundance of at least 0.1%. For statistical analysis of alpha diversity, Chao1 measure was used at the Feature level of classification. For two class comparisons, a *t* test was used. For three or more class comparisons an ANOVA test was used. A *P* value threshold of less than 0.05 was used for identification of significant results. For univariate analysis of OTUs, the Feature level of classification was used. For two class comparisons, a *t* test was used. For three or more class comparisons an ANOVA test was used. A *P* value threshold of less than 0.05 was used for identification of significant results. For beta diversity profiling, Principal Coordinate Analysis (PCoA) was used using the Bray-Curtis index for distance measure. The significance of beta diversity modelling was testing using the PERMANOVA function with a significant threshold of less than 0.05.

**Metabolic Fingerprinting using Laser Assisted Rapid Evaporative Ionisation Mass Spectrometry (LA-REIMS)**

The laser was operated at 3.5 W in 40 ms pulses (40 ms on followed by 40 ms off) with the pulsatile SuperPulse mode used to deliver the majority of the energy at the beginning of each pulse. Each sample was heated for 8 seconds with the laser spot moving in a figure-of-eight pattern over an area of approximately 0.8 mm^2^. Heating with the CO2 laser results in a vapour containing complex lipids and low molecular weight metabolites (typically below 2.5 kDa) in gas-phase. This analyte-containing vapour was then aspirated towards a REIMS interface (Waters Corporation, Wilmslow, UK) attached to a Xevo G2-XS quadrupole time-of-flight mass spectrometer (QToF-MS) using the instrument’s native vacuum. Within the REIMS interface (Waters Corporation), the analyte-containing vapour mixes with a 0.25 mL/minute infusion of 2-propanol (Romil, Cambridge, UK) containing leucine enkephalin (Sigma-Aldrich, Gillingham, UK) at 0.01 ng/µL as external lock mass compound. This mixture then collides with a kanthal ribbon heated to ≈ 900°C which acts to break apart ion clusters prior to mass spectrometry analysis. Samples were analysed in negative and positive ion detection modes in separate runs between which the samples were mixed and subjected to a second centrifugation. The mass spectrometer was operated in sensitivity mode with scans collected every second. Prior to running, the instrument was calibrated using sodium formate in both polarities according to the manufacturer’s instructions.

**Processing and Statistical Analysis of LA-REIMS Metabolic Fingerprinting Data**

Raw data for individual samples was imported into R studio (version 3.4.4) using a modified Waters Corporation raw file reader (Waters Corporation) and processed to remove background signal and correct for mass drift according to the monoisotopic mass of leucine enkephalin ([M-H]- mass 554.2615 and [M-H]+ mass 556.2712). From the processed spectra, individual masses were picked using the MALDIquant package to a four decimal place accuracy as a consensus across all samples in which that feature was detected. A data matrix was created following total ion count normalisation and used in subsequent data analysis. For statistical analysis using the MetaboAnalyst 3.0 pipeline [6], each data matrix was subjected to Log_10_ transformation and Pareto scaling after removal of low-variance variables based on standard deviation. A statistical cut-off of false discovery rate corrected *P* values in one-way analysis of variance univariate analysis was < 0.05 and class differences were identified using Fisher’s LSD *post hoc* analysis. Tentative metabolite identifications are obtained from the Human Metabolome Database [7] and LIPIDMAPS [8] database using a search threshold of mass accuracy below 10 ppm. Where multiple potential matches were given, the [M-H]+ or [M-H]- ion was chosen over other matches where available. If not available, the match with the lowest ppm value was chosen as tentative identification after removal of metabolites associated with health status or use of pharmaceuticals.

**Figure S1. Negative extraction controls show no biologically meaningful contamination**

Negative extractions controls of PCR grade water were included in each extracted batch of 23 human milk samples. Significant differences were observed following analysis of both (a) Chao1 alpha-diversity index (*t* test *P* value below 0.05), and (b) multivariate beta-diversity show significant separation between blank extraction negative controls and human milk samples (PERMANOVA *P* value below 0.05). Blank extraction samples are shown as red data points and extracted human milk samples are shown as blue data points. Figure was created using the MicrobiomeAnalyst platform as detailed in the supporting methods section using raw reads as rarefaction would have biased analysis based on low number of reads in blank extraction controls. A total of six negative extraction controls and 46 human milk samples were used in figure construction.


**Figure S2. Correlation analysis of microbiota features shows co-occurring clusters**

Correlation analysis of human milk samples across all age ranges suggests strong correlations between certain microbiota features in co-occurring clusters. Correlation was complete using Pearson’s correlation coefficient with a Bonferroni adjusted *P* value threshold of < 0.05. Self-correlations between microbiota features were removed. Size of dot indicates size of correlation coefficient with only positive correlations emerging as significant. Colour coding indicates total number of correlations for that microbiota features on the y-axis. A total of 46 replicates were used in correlation analysis.

**Figure S3. REIMS is capable of clear differentiation between human and bovine-derived formula**

Principal component analysis of human milk and bovine-derived commercially available ready-made formula milk for three age ranges. Clear separation is seen between human milk and formula in both (a) negative ion detection and (b) positive ion detection REIMS modalities. Figure was created using the MetaboAnalyst pipeline as detailed in the supporting methods information for metabolic fingerprinting analysis using total ion current normalised, glog transformed and Pareto scaled data. A total of 72 formula milk replicates and 62 human milk replicates were used in figure construction.

**Figure S4. Chemical taxonomy classification of identified REIMS metabolite features**

REIMS features were identified using HMDB and LIPID MAPS databases with a mass tolerance of <10 ppm. Chemical taxonomy classifications are shown at the (a) kingdom and (b) class level to support the class level classification in main manuscript. Features with no match were removed from sub-kingdom classification searches. The number of features identified in negative ion detection mode are shown in pink (left) and positive ion detection mode in blue (red).

**Figure S5. Univariate statistics suggest significant differences may result from concentration**

Based on false discovery rate (FDR)-corrected ANOVA results, using a corrected *P* value threshold of less than 0.05, the output of MetaboAnalyst was tabulated to determine higher or lower levels in statistical differences. Class differences were identified using Fisher’s LSD *post hoc* analysis. For each significant metabolite feature, blue shading in nursling age group column indicates feature has a significantly higher intensity than at least one other nursling age group. If no blue shading is present, that feature does not have a significantly higher intensity than any other nursling age group. Labelling on y-axis indicates negative or positive ion detection mode, detected molecular weight of metabolite feature, and tentative identification of feature based on information available in Supplementary Data Matrix 2. A total of 62 human milk replicates were used in univariate analysis.

**Figure S6. PLSDA modelling of expression method and nursling gender as potential confounders**

Supervised PLSDA modelling of REIMS metabolite fingerprinting for method of milk expression for (a) REIMS negative ion detection mode and (b) REIMS positive ion detection mode shows no significant differences. Modelling of nursling gender for both (c) REIMS negative ion detection mode and (d) REIMS positive ion detection mode shows no significant differences. A total of 62 human milk replicates were used for figure construction.

**Supporting References**

1. Bolyen, E.; Rideout, J.R.; Dillon, M.R.; Bokulich, N.A.; Abnet, C.; Al-Ghalith, G.A.; Alexander, H.; Alm, E.J.; Arumugam, M.; Asnicar, F. *QIIME 2: Reproducible, interactive, scalable, and extensible microbiome data science*; 2167-9843; PeerJ Preprints: 2018.

2. Callahan, B.J.; McMurdie, P.J.; Rosen, M.J.; Han, A.W.; Johnson, A.J.A.; Holmes, S.P. DADA2: high-resolution sample inference from Illumina amplicon data. *Nature methods* **2016**, *13*, 581.

3. Schmieder, R.; Edwards, R. Quality control and preprocessing of metagenomic datasets. *Bioinformatics* **2011**, *27*, 863-864.

4. DeSantis, T.Z.; Hugenholtz, P.; Larsen, N.; Rojas, M.; Brodie, E.L.; Keller, K.; Huber, T.; Dalevi, D.; Hu, P.; Andersen, G.L. Greengenes: A Chimera-Checked 16S rRNA Gene Database and Workbench Compatible with ARB. *Applied and Environmental Microbiology* **2006**, *72*, 5069-5072, doi:10.1128/AEM.03006-05.

5. Dhariwal, A.; Chong, J.; Habib, S.; King, I.L.; Agellon, L.B.; Xia, J. MicrobiomeAnalyst: a web-based tool for comprehensive statistical, visual and meta-analysis of microbiome data. *Nucleic acids research* **2017**, *45*, W180-W188.

6. Xia, J.; Sinelnikov, I.V.; Han, B.; Wishart, D.S. MetaboAnalyst 3.0 - Making Metabolomics More Meaningful. *Nucleic Acids Research* **2015**, *43*, W251-257, doi:10.1093/nar/gkv380.

7. Wishart, D.S.; Jewison, T.; Guo, A.C.; Wilson, M.; Knox, C.; Liu, Y.; Djoumbou, Y.; Mandal, R.; Aziat, F.; Dong, E., et al. HMDB 3.0: The Human Metabolome Database in 2013. *Nucleic Acids Research* **2013**, *41*, D801-807, doi:10.1093/nar/gks1065.

8. Fahy, E.; Subramaniam, S.; Murphy, R.C.; Nishijima, M.; Raetz, C.R.H.; Shimizu, T.; Spener, F.; van Meer, G.; Wakelam, M.J.O.; Dennis, E.A. Update of the LIPID MAPS comprehensive classification system for lipids. *Journal of Lipid Research* **2009**, *50*, S9-S14, doi:10.1194/jlr.R800095-JLR200.

**Supplementary Data Matrix 1 – Link Anonymised Individual Participant and Sample Information**

Microsoft Excel spreadsheet (.xlsx) file contains link anonymised individual participant and sample information that is used and referenced through the main manuscript and supporting data.

**Supplementary Data Matrix 2 – Tentative Identification of REIMS Detectable Human Milk Components**

Microsoft Excel spreadsheet (.xlsx) file contains tentative identifications of all detectable human milk components from the Human Metabolome Database and LIPIDMAPS database as detailed in the supplementary methods section for bioinformatic analysis of REIMS metabolic fingerprinting data. One sheet details the tentative identifications for negative ion detection mode data, and one for positive ion detection mode data. The experimental *m/z* value is given alongside the matched database value and the corresponding ppm error value. A reference to the database identification is given for HMDB or LIPIDMAPS databases. Chemical classifications are based on the structural hierarchy used in HMDB. Where multiple possible matches are possible based on ppm of matches, an asterix is marked in the ‘Multiple’ column to indicate that structural isomers cannot be differentiated.
